# How Prepared Was Pakistan for the COVID-19 Outbreak?

**DOI:** 10.1017/dmp.2020.247

**Published:** 2020-07-14

**Authors:** Muhammad Salman, Zia Ul Mustafa, Tahir Mehmood Khan, Naureen Shehzadi, Khalid Hussain

**Affiliations:** Faculty of Pharmacy, The University of Lahore, Lahore, Pakistan; District Headquarter Hospital, Pakpattan, Pakistan; Institute of Pharmaceutical Science, University of Veterinary and Animal Sciences, Lahore, Pakistan; School of Pharmacy, Monash University, Bandar Sunway, Selangor, Malaysia; Punjab University College of Pharmacy, University of the Punjab, Lahore, Pakistan

**Keywords:** China, coronavirus, outbreak, Pakistan

The newly emerged coronavirus, severe acute respiratory syndrome coronavirus 2 (SARS-CoV-2), now a pandemic, is spreading far more rapidly than the earlier coronaviruses, as well as other infectious disease outbreaks of the recent past. As effective preparedness is pivotal to significantly minimize damage from imminent and unexpected disaster, here, we critically analyzed Pakistan’s preparedness for the coronavirus disease (COVID-19).

China is located northeast of Pakistan and both countries share more than a 500-kilometer border. The people of both these countries travel frequently due to multiple economic ties, technical assistance of the China Pakistan Economic Corridor, studies, and tourism. Hence, the likelihood of a COVID-19 outbreak in Pakistan was immensely high. In late January 2020, according to the news, 5 individuals (1 Pakistani and 1 Chinese in Multan and 3 Chinese in Lahore) who recently came from China were suspected of COVID-19 and hospitalized. This led to fear and chaos in the public. The National Institute of Health, Pakistan, issued an advisory (F.1-22/Advisory/FEDSD/2020) regarding the disease, which was endorsed by the Director of General Health, and an alert was issued (443-523/PA/DGHS) to all the hospitals of Punjab Province asking staff members to remain vigilant about any suspected cases. However, this alone was not enough to effectively tackle the impending SARS-CoV-2 attack in Pakistan. In our opinion, the following measures should have been implemented by the health regulators.

1. Pakistanis should have been advised to avoid non-essential travels to the disease-struck areas.
2. Individuals coming from COVID-19-infested countries should have been properly screened and tested at entry points.
3. Properly equipped quarantine facilities should have been established near entry points on the borders.
4. Pakistani health professionals must be comprehensively educated about the SARS-CoV-2. Moreover, the procurement and supply of diagnostic kits, personal protective equipment, as well as adequate amount of life-saving equipment should be ensured.
5. Information of the disease, such as symptoms, mode of transmission, preventive measures (hand hygiene, respiratory hygiene/cough etiquette, social distancing), and treatment about the disease should have been provided to the general public.

Unfortunately, the health regulators were slow to act on COVID-19. It was taken seriously for the first time toward the end of January 2020, and screenings were started at major airports.^1^ However, screening at all border entry points were not initiated until after the first 2 positive-tested patients (February 26, 2020) came from Iran – Pakistan’s neighbor to the west, one of the most severely affected countries with COVID-19. This led to thousands of pilgrims coming from Iran to be quarantined at camps without proper arrangements (no real housing, poor sanitation, lack of basic preventive measures, lack of proper testing and medical facilities), making the matter worse.^2^ Due to the failure of border quarantines, provinces were compelled to prepare their own quarantine facilities to house the pilgrims, which did not work out well either and so local viral transmissions began. Soon, owing to the insufficient provision of personal protective equipment, Pakistani health professionals started protesting, even going on a hunger strike and threatening to quit work.^3-5^ Furthermore, awareness campaigns in the country commenced late (March 13, 2020). The vague policies resulted in an alarming high rate of disease spread until June 8, 2020; there were 103 671 COVID-19 cases in the country (67 249 active cases, 2067 deaths, and 34 355 recoveries).^6^ In conclusion, Pakistan’s preparedness for COVID-19 was far from satisfactory. The health regulators still need to devise and implement a clear policy to effectively combat COVID-19.
